# Continuous bidirectional coupling of heart rate variability and emotions in dyad speaker-listener dynamics reveals unique interpersonal synchronization

**DOI:** 10.3389/fnsys.2026.1802891

**Published:** 2026-05-29

**Authors:** Mohanad Alkhodari, Ioannis Ziogas, Jaskeerat Singh, Paolo Grigolini, Glenn W. Muschert, Fedor Kusmartsev, Herbert F. Jelinek

**Affiliations:** 1 Department of Medical Sciences, Khalifa University, Abu Dhabi, United Arab Emirates; 2 Cardiovascular Clinical Research Facility (CCRF), Radcliffe Department of Medicine, University of Oxford, Oxford, United Kingdom; 3 Department of Biomedical Engineering & Biotechnology, Khalifa University, Abu Dhabi, United Arab Emirates; 4 Center for Nonlinear Science, University of North Texas, Denton, TX, United States; 5 Healthcare Engineering Innovation Group (HEIG), College of Medicine and Health Sciences, Khalifa University, Abu Dhabi, United Arab Emirates; 6 Department of Public Health and Epidemiology, Khalifa University, Abu Dhabi, United Arab Emirates; 7 Department of Physics, Khalifa University, Abu Dhabi, United Arab Emirates

**Keywords:** bidirectional coupling, emotional dynamics, entropy and complexity, heart rate variability, physiological synchronization

## Abstract

**Background:**

Human conversation involves moment-to-moment reciprocal adjustments between interlocutors, expressed through both emotional cues and autonomic physiology.

**Objectives:**

To quantify how physiological synchrony continuously builds and subsides between debate partners during speaker-listener turn-taking, and to test whether the direction of this coupling (speaker-leading vs listener-leading) is associated with (i) self- versus partner-perceived arousal/valence and (ii) autonomic and complexity-based heart rate variability (HRV) characteristics.

**Methods:**

Multimodal data from the K-EmoCon database were analyzed, comprising HRV-derived cardiac activity, speech timing, and multi-perspective emotion ratings from 32 individuals engaged in a structured dyadic debate. Interactions were segmented into speaking and listening phases, and a phase-based bidirectional coupling framework was applied to quantify both the strength and polarity of physiological synchrony. Associations between emotional states and HRV features were examined using correlation analysis across coupling segments, followed by principal component analysis (PCA), to reduce dimensionality and cluster emotions and features based on their shared variance.

**Results:**

Positive coupling segments, corresponding to speaker-leading dynamics, were characterized by strong associations between partner-related emotional states and parasympathetic HRV indices, including RMSSD and SD1, with correlations reaching up to 0.63 (*p* < 0.001). In contrast, negative coupling segments, reflecting listener-leading dynamics, showed stronger associations with sample entropy, Rényi entropy, and low-frequency power, with correlations reaching 0.71 (*p* < 0.001). Diffusion entropy exhibited a polarity-dependent pattern consisting of positively correlated self-reported emotions during positive coupling, whereas during negative coupling it was negatively correlated with partner-related emotions, with correlations reaching 0.71 (*p* < 0.001) for the complexity index μ_*r*_ at scale 1. PCA showed that positive coupling was characterized by a clear separation of arousal, with self-related emotions aligning with diffusion entropy features and partner-related emotions clustering with HRV and Rényi entropy measures. In contrast, negative coupling exhibited a pattern in which partner-related emotions formed more compact clusters across power- and entropy-based features.

**Conclusions:**

These findings demonstrate that bidirectional physiological coupling provides a sensitive framework for disentangling leadership, responsiveness, and emotional exchange during conversation. By revealing distinct autonomic and complexity-based signatures of self- and partner-related affect, this work advances understanding of interpersonal emotional regulation. It has implications for therapeutic, educational, and collaborative communication contexts.

## Introduction

Bidirectional coupling of physiological features during conversations has been shown to be related to the continuous, dynamic exchange and two-way transmission of verbal information between participants (Schoot et al., 2016). Bidirectional coupling refers to the continuous, reciprocal interaction between two individuals’ physiological signals, in which each participant both influences and is influenced by the other over time. In this process, not only is speech perceived and produced, but mutual synchronization also emerges between conversational partners in their physiological responses and emotional states (Lin et al., 2024). Through bidirectional coupling, speakers and listeners influence one another in real time, often through gestures, speech tone, facial expressions, and subtle timing cues (Hess et al., 2020; Jiang et al., 2021).

Such reciprocal interaction highlights conversation as a dynamically coordinated process involving continuous mutual adjustment between participants (Alhussein et al., 2025). From a systems neuroscience perspective, interpersonal interaction can be seen as a coupled dynamical system involving both central neural circuits and peripheral autonomic regulation (de Zambotti et al., 2018). Brain regions involved in socio-affective processing continually integrate internal physiological signals with external social cues (Antonelli et al., 2025). These systems facilitate predictive regulation of oneself and others, supporting coordinated changes in behavior, emotion, and physiology. In this context, physiological measures offer valuable insight into the psychological processes observed during social interaction. Signals such as electrocardiography (ECG), photoplethysmography (PPG), and derived heart rate variability (HRV) have been widely used to investigate stress, attentional engagement, and affective dynamics during conversational exchanges (Alhussein et al., 2023, 2025; Armañac-Julián et al., 2025; Divjak et al., 2024). HRV indices provide a measure of the dynamic interplay between sympathetic and parasympathetic branches of the autonomic nervous system and are commonly regarded as a robust indicator of emotional regulation and adaptive capacity (Alkhodari et al., 2024). In parallel, emotions are strongly influenced by physiological changes and continuously evolve and influence the conversational tone between partners (Wang et al., 2023).

A growing body of research has examined physiological and neural synchrony during interpersonal interaction, demonstrating its relevance to engagement, emotional alignment, and social coordination (Birchler et al., 2024; Chatterjee et al., 2025). Prior studies have explored synchrony using measures derived from heart rate variability, electrodermal activity, and neural signals across various contexts, including psychotherapy, collaborative learning, and naturalistic conversations (Chatterjee et al., 2021; Dikker et al., 2017; Helm et al., 2018; Tschacher and Meier, 2020). These works have shown that synchrony is associated with interpersonal engagement, therapeutic alliance, and shared attention. Moreover, various methods have been proposed to quantify physiological synchrony, including correlation-based approaches, spectral coherence, phase-locking value (PLV), and phase-lag index (PLI) (Helm et al., 2018; Jiang et al., 2015; Konvalinka et al., 2014). However, most existing approaches focus primarily on the magnitude of synchrony or rely on unidirectional or symmetric measures, with limited consideration of bidirectional and continuous lead-lag dynamics and their relationship to emotional exchange.

**FIGURE 1:**
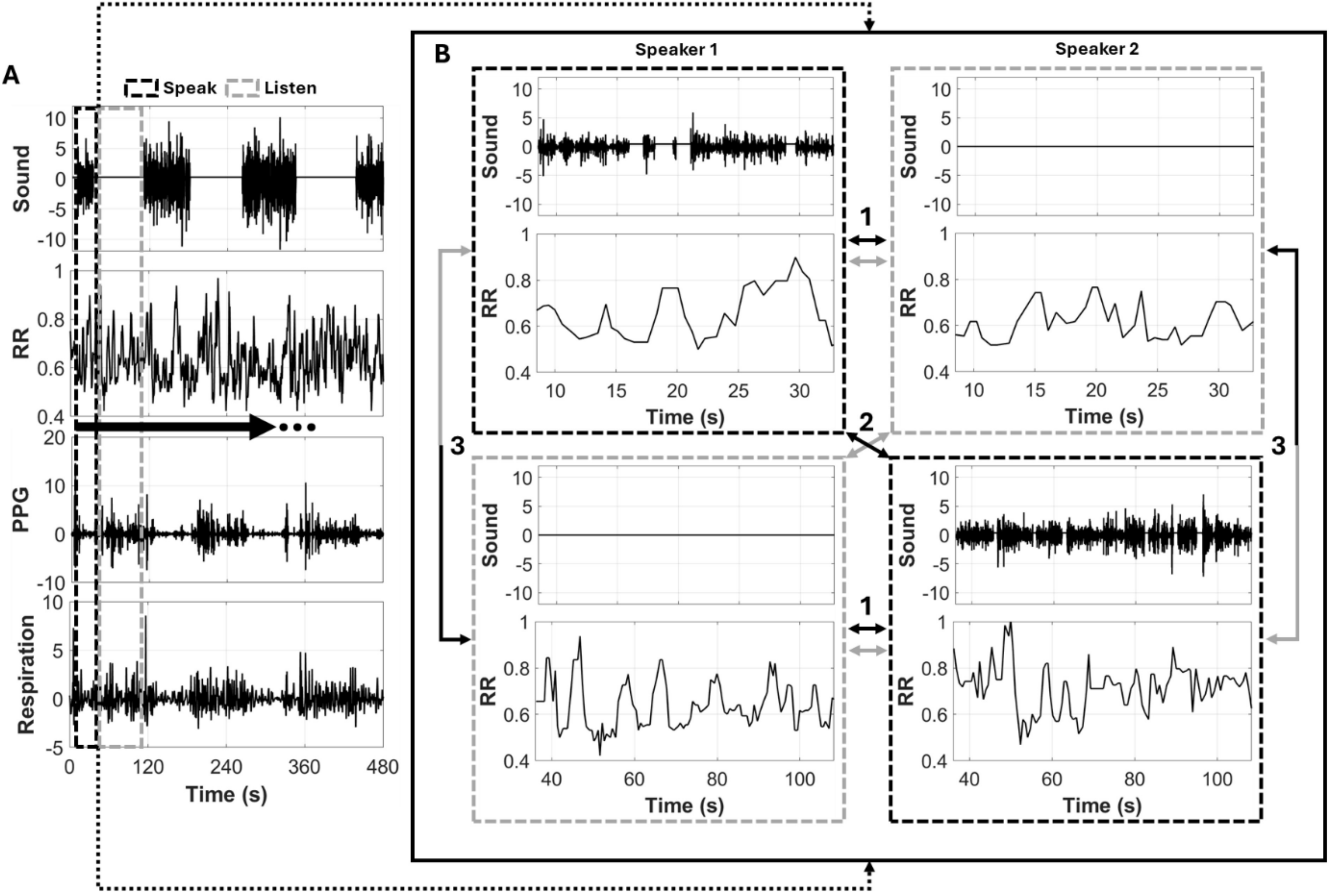
Overview of the interpersonal coupling analysis pipeline. Schematic of the conversational setting in the K-EmoCon dataset and the types of data collected. **(A)** The example, drawn from group 9 (participant 1), illustrates speech activity alongside the corresponding physiological signals. The signals are segmented into speaking (black) and listening (gray) periods. **(B)** A magnified view of the speaking-listening analysis highlights three main phases: (1) simultaneous coupling, (2) cross-participant coupling, and (3) within-participant coupling.

In light of this, we examined continuous, bidirectional physiological coupling in dyadic conversation by integrating time-aligned cardiac, speech, and emotion data from the K-EmoCon dataset. Specifically, we (1) propose a novel complex-valued phase-based coupling index that captures both coupling strength and lead-lag polarity, thereby simultaneously characterizing temporal directionality of interpersonal coupling, (2) separated coupling into positive (speaker-leading) and negative (listener-leading) segments, and (3) test how these polarity-defined segments map onto self-, partner-, and external emotion ratings and autonomic/complexity HRV features. In this study, we only focus on the polarity of the coupling to distinguish between speaker-leading and listener-leading dynamics during interaction. Finally, we examined the real component of the coupling vector to distinguish between magnitude-driven synchrony and in-phase alignment. This framework incorporates information on whether the speaker or listener leads at any time during the debate, and which physiological characteristics accompany interpersonal emotional exchange (Figure 1).

## Materials and methods

### Dataset

The study utilized data from the publicly available K-EmoCon multimodal database (Park et al., 2020), which comprises English-language dyadic debates on the socially sensitive topic of accepting refugees from Yemen in Korea. The dataset includes 32 participants (aged 23–36), randomly paired into 16 dyads, with each debate session lasting approximately 10 min. The conversations consisted of structured dyadic debates on the socially sensitive topic of refugee immigration. During each session, participants were assigned opposing viewpoints and engaged in a structured debate format. A moderator introduced the topic and oversaw the discussion to maintain its structure and timing, occasionally intervening to facilitate balanced participation between speakers, but did not actively contribute to the debate content itself. To capture multimodal emotional expression, the database provides photoplethysmography (PPG) signals alongside synchronized audio recordings of speech and video recordings of facial expressions and body movements.

### Physiological signals

Physiological features were derived from PPG signals acquired using the Empatica E4 wristband at a sampling frequency of 64 Hz. The recordings were temporally trimmed according to the session start and end times specified in the metadata to ensure alignment across participants. To reduce noise, raw PPG signals were filtered using a fourth-order zero-phase Butterworth band-pass filter with cut-off frequencies of 0.5–5 Hz, retaining the frequency range associated with PPG variability while attenuating motion-related artifacts and baseline drift (Lapitan et al., 2024).

Cardiac pulse peaks were subsequently identified from the filtered PPG to derive beat-to-beat intervals (RR intervals). Peak detection was implemented using MATLAB’s *findpeaks* function with a minimum peak separation of 0.4 s, corresponding to a maximum plausible heart rate of 150 beats per minute. To mitigate noise and remove erroneous detections, the resulting intervals were further processed using the SDROM algorithm, following the procedure outlined in our previous work (Alkhodari et al., 2021b; Saleem et al., 2022). The cleaned RR intervals were then interpolated onto the continuous PPG time axis to obtain frame-level RR estimates aligned with the original recordings. In addition, heart rate (HR) was computed continuously and incorporated into the feature adjustment process via linear regression to account for HR-related variability and effects.

Respiratory activity was estimated from the PPG signals using the method proposed by Charlton et al. (2017). Specifically, three respiration-related components were derived: baseline wander, capturing low-frequency fluctuations in the PPG baseline due to thoracic motion; amplitude modulation, extracted using the Hilbert envelope to reflect respiration-driven changes in pulse amplitude; and frequency modulation, representing respiratory sinus arrhythmia observed in beat-to-beat interval variations. Each component was band-pass filtered within the typical respiratory frequency range of 0.1–0.7 Hz (Leiva et al., 2025), normalized, and subsequently averaged to produce a single composite respiration signal. From this signal, the respiratory rate was estimated and used to further adjust the extracted features through linear regression, thereby reducing respiration-related confounding effects.

### Speech recordings

Debate audio recordings underwent a dedicated preprocessing pipeline to incorporate physiological measures alongside speech (Ziogas et al., 2024). As the K-EmoCon dataset was collected in naturalistic conversational environments, the raw audio included overlapping speech, moderator interruptions, and various background noise sources. To address these challenges, a preprocessing strategy emphasizing source separation was employed by us in this study. Audio signals were first normalized via max-scaling, followed by the removal of silent segments using short-time Fourier transform (STFT)-based energy thresholding. Speech-containing segments were then manually annotated and denoised using either the WTST-NST filtering method to suppress the stationary background noise (Hadjileontiadis et al., 2000) or a pre-trained Sepformer model (trained on the LibriMix dataset) to separate overlapping voices, depending on the characteristics of the noise present (Subakan et al., 2023).

Following this preprocessing step, the cleaned audio data were prepared for joint analysis with the physiological signals. Segment annotations were used to split each debate into speaker-specific audio tracks, which were subsequently resampled and temporally aligned from the original 22.5 kHz audio sampling rate to the 64 Hz sampling frequency of the physiological signals, including PPG, RR intervals, and respiration. From this synchronized representation, two categories of speech-related features were extracted: (i) binary indicators of speaking activity (speak1, speak2), identifying whether each participant was speaking at a given time frame, and (ii) continuous vocal intensity measures (sound1, sound2), reflecting the relative amplitude of each speaker’s speech. This alignment procedure produced debate-level speech features that were temporally synchronized with both physiological measurements and emotional annotations.

### Emotional states

Emotional annotations were collected at 5-s intervals from three complementary sources: self-reports from speakers, partner-based evaluations from listeners, and third-person assessments from five independent external raters. These annotations were mapped onto the continuous affective dimensions of arousal and valence, each rated on a five-point scale (1–5), enabling fine-grained analysis of emotional dynamics over time. Following the annotation protocol of the K-EmoCon dataset, participants retrospectively reviewed recordings of the debate after the interaction and provided ratings of their own emotional state as well as their perception of their partner’s emotional state at each 5-s segment. Self-arousal and self-valence therefore represent the participant’s own reported emotional intensity and affective valence, whereas partner-arousal and partner-valence represent the participant’s perception of their conversational partner’s emotional state. External annotations were provided independently by five raters and aggregated in the dataset by averaging their scores. In the present study, these measures were treated as continuous variables reflecting the temporal variation of arousal and valence throughout the interaction.

To align the discrete emotion labels in the K-EmoCon dataset with the continuously sampled physiological recordings, we temporally expanded each annotation, originally provided at 5-s intervals, to cover its corresponding duration and replicated across all frames within that segment. The annotation timestamps, expressed in seconds, were directly mapped to the relative timeline of the PPG recordings to ensure accurate temporal correspondence. This up-sampling process produced frame-level arousal and valence sequences synchronized with the 64 Hz sampling frequency of the physiological data. For the external emotion evaluations, we employed the aggregated labels in the dataset, derived by averaging the ratings from five independent annotators. The up-sampling does not introduce new emotional information but ensures temporal alignment with physiological signals.

### Feature extraction

**FIGURE 2:**
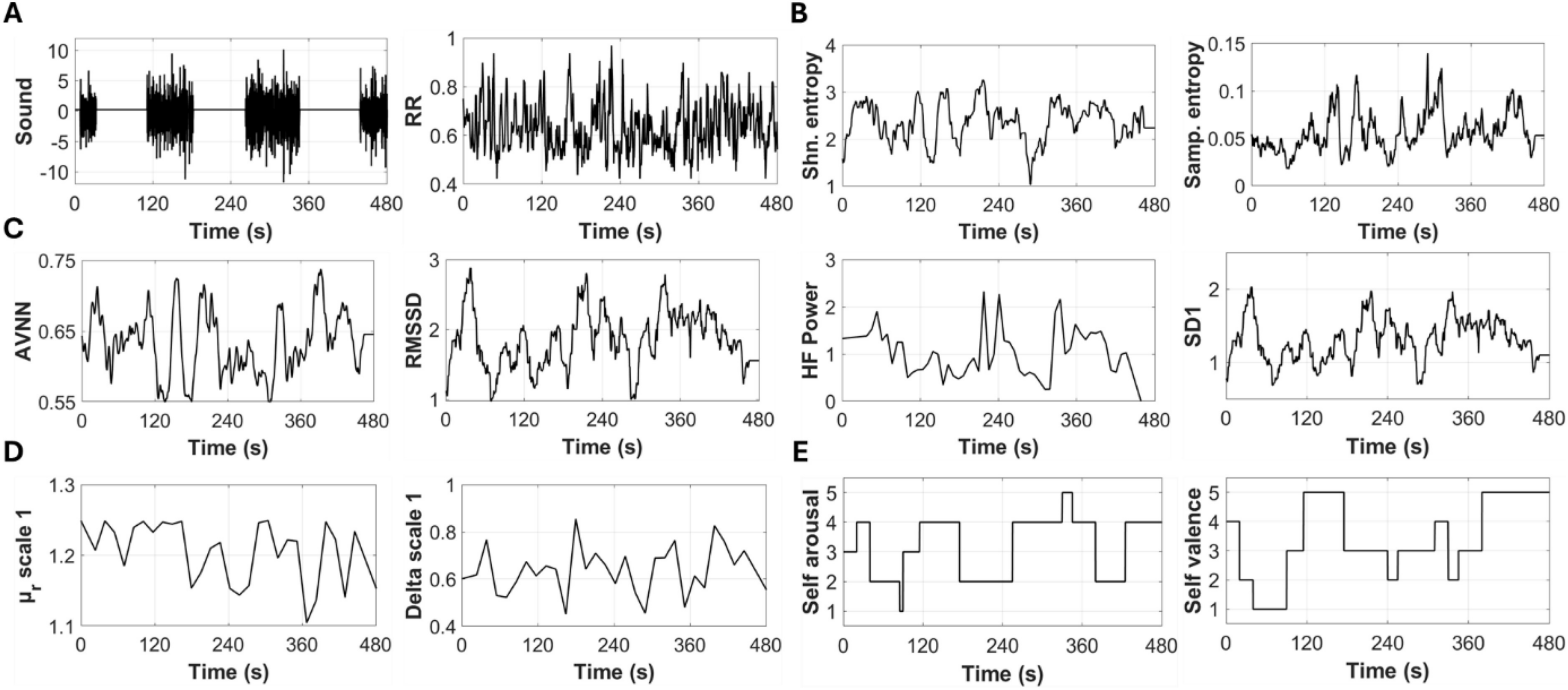
Features derived from peak-to-peak (RR) intervals. The extracted RR-based measures reflect alignment with emotional dimensions (self arousal and valence) and include Shannon entropy, sample entropy, diffusion entropy, and heart rate variability (HRV) metrics. Showing examples of: **(A)** speech sound and RR interval, **(B)** Shannon and sample entropies, **(C)** heart rate variability (HRV) features, **(D)** diffusion entropy feature, and **(E)** emotional states for self annotations.

To quantify signal complexity and enable comparative analyses, information-theoretic metrics were extracted from RR interval signals. Feature extraction was performed using sliding windows of 1,000 samples with a one-sample increment, yielding continuous feature vectors that were temporally synchronized with the original time series. Examples of these features are provided in Figure 2.

Shannon entropy was computed to estimate the average informational content of each signal’s probability distribution, providing an index of global unpredictability (Feutrill and Roughan, 2021). To capture temporal irregularity, sample entropy was calculated by assessing the probability that similar patterns in the time series remain similar when extended in time, using parameters m = 2 and *r* = 0.2 (Patel et al., 2021). In addition, Rényi entropy was employed as a generalized entropy measure that extends Shannon’s formulation by introducing a tuneable parameter α, allowing differential weighting of rare versus frequent events (Cornforth et al., 2013). Rényi entropy was evaluated across multiple scales (α∈ {-5, -3, -1, 1, 3, 5}), where negative α values increase sensitivity to low-probability events, positive values emphasize high-probability events, and α = 1 converges to the Shannon entropy. To ensure consistency across time windows, all entropy measures were computed using a global histogram with ten bins per recording.

Beyond single-scale entropy measures, multiscale diffusion entropy analysis (MSDEA) was applied following the methodology proposed by Nasrat et al. (2023) to examine the temporal dynamics of the signals. The time series were coarse-grained at progressively increasing dyadic scales (4, 8, 16, 32, and 128) using block averaging, and diffusion entropy was estimated for each scale. Two diffusion-based descriptors were subsequently derived in accordance with the framework introduced by Benfatto et al. (2021), Jelinek et al. (2021): the scaling exponent (δ), which captures the rate of entropy growth over diffusion time, and the complexity index (μ_*r*_), computed from δ and reflecting the intrinsic dynamical complexity of the signal.

From the RR interval sequences, a comprehensive set of conventional heart rate variability (HRV) features was also extracted (Alkhodari et al., 2021a,b). Time-domain metrics included the mean RR interval (AVNN), standard deviation of RR intervals (SDNN), root mean square of successive differences (RMSSD), the proportion of successive intervals differing by more than 50 ms (pNN50), and the standard error of the mean (SEM). Frequency-domain features were derived from power spectral density estimates obtained using Welch’s method with overlapping segments. They included absolute and normalized power in the very-low-frequency (VLF), low-frequency (LF), and high-frequency (HF) bands, peak frequencies within each band, total spectral power, and the LF/HF ratio. Frequency-domain features were calculated on a 3,840-sample moving window, corresponding to a 60-s segment at the 64 Hz sampling frequency, thereby providing sufficient frequency resolution for reliable estimation using the Welch method. Finally, nonlinear characteristics were quantified using Poincaré plot descriptors (SD1 and SD2) and detrended fluctuation analysis (DFA).

### Interpersonal speak-listen scenarios

To examine interpersonal coupling, we analyzed participants’ RR intervals and speech signals during debate segments defined by speaking and listening states (Figure 1). Segments were identified using binary speech indicators and analyzed independently to maintain interaction timing. Coupling was evaluated from three perspectives: (1) simultaneous coupling when one participant spoke and the other listened, (2) cross-participant coupling when both participants were in the same state (speaking or listening), and (3) within-participant coupling by comparing each individual’s RR activity during speaking versus listening.

### Analysis of bi-directional coupling

Within each speaking/listening segment, we propose a new coupling index that computed the instantaneous phase difference between participants’ band-limited RR signals and derived a complex-valued bidirectional coupling index (Bi λ) (Jelinek et al., 2025). The magnitude of Bi λ reflects phase-locking strength. At the same time, the sign/polarity of the mean phase difference indicates lead–lag direction: Bi λ > 0 denotes Participant 1 leading (speaker-leading in simultaneous segments) and Bi λ < 0 denotes Participant 2 leading (listener-leading in that ordering).

Coupling information was extracted between two segments as an extension to the conventional uni-directional phase analysis (Jelinek and Khandoker, 2020) forming a bi-directional phase coupling degree as follows:


$$\lambda \,\left(t_{k}\right)=|\frac{1}{N}\,\overset{k+\frac{N}{2}}{\underset{k-\frac{N}{2}}{\sum }}e^{\left[\phi_{1}\,\left(t_{k}\right)-\phi_{2}\,\left(t_{k}\right)\right]\,m\,o\,d\,2\,\pi }{|}^{2}$$

where *k* denotes the time step in *N* overall lengths of the selected signals and φ_1(t_k_) and φ_2(t_k_) are the instantaneous phases of the two RR segments obtained using the Hilbert transform. We calculated the Bi λ(t_k_) using the phase coupling degree of synchronization (Jelinek and Khandoker, 2020) as follows:


$$B\,i\,\lambda \,\left(t_{k}\right)=\lambda \,\left(t\,k\right)\times \tan^{-1}\left(\frac{\cos\left(\lambda \,\left(t_{k}\right)\right)}{\sin\left(\lambda \,\left(t_{k}\right)\right)}\right)$$

where Bi λ ranges from −1 to 1. In this specific scenario, positive coupling indicates first-segment interaction, while negative coupling indicates second-segment interaction.

Retaining the complex-valued coupling vector addresses the conventional applied reductions, e.g., taking only the cosine of the phase difference, that can spuriously inflate synchrony estimates and remove directional information. Previous results in neurophysiology have shown that measures explicitly sensitive to non-zero phase lags, such as the imaginary part of coherency and the phase-lag index, improve interpretability by discounting instantaneous (zero-lag) coupling (Stam et al., 2007). Analogously, the current decomposition application into magnitude, real part, and sign/polarity preserves biologically meaningful lead-lag structure during interaction (Vinck et al., 2011).

Because the Bi λ angle is strongly influenced by the imaginary component of the complex coupling vector, we also examined the real component to better disentangle coupling strength from phase directionality. Both elements can be obtained as follows,


$$C_{r\,e\,a\,l}\,\left(t_{k}\right)=\frac{\cos\left(\left[\phi_{1}\,\left(t_{k}\right)-\phi_{2}\,\left(t_{k}\right)\right]\,m\,o\,d\,2\,\pi \right)}{N}$$

$$C_{i\,m\,a\,g}\,\left(t_{k}\right)=\frac{\text{sin}\,\left(\left[\phi_{1}\,\left(t_{k}\right)-\phi_{2}\,\left(t_{k}\right)\right]\,m\,o\,d\,2\,\pi \right)}{N}$$

This decomposition enabled complementary analyses of magnitude-dominated synchronization (real part) besides the phase-lead/lag asymmetries (imaginary part), providing a more nuanced characterization of coupling dynamics.

The polarity of the bidirectional coupling index indicates dynamic shifts in which the individual exerts regulatory control within the dyad, rather than just phase lead–lag relationships. Positive coupling (speaker-leading) likely represents top-down modulation related to communicative intent and expressive control, whereas negative coupling (listener-leading) suggests heightened responsiveness to external inputs and adaptive updating depending on the magnitude of the coupling. This view supports hierarchical control models in which central neural systems switch flexibly between generative (output-focused) and receptive (input-focused) modes during social interactions.

### Analysis of correlation

To examine the relationships among emotion states, extracted features, and coupling measures, we computed Pearson correlation coefficients between each emotion dimension (arousal and valence) at each perspective (self, partner, and external) and features across all participants. Correlation analysis was performed separately for positive and negative regions based on the Bi λ. The positive and negative Bi λ regions were tracked, and their corresponding times, feature values, and emotional states were aggregated accordingly across all speak-listen segments in the three phases. The analysis was performed on aggregated segments rather than individual time points, and results are interpreted in terms of relative patterns across conditions rather than absolute pointwise associations. The resulting correlation coefficients were calculated and further visualized using heatmaps, providing an overview of associations across all emotion-feature combinations. To highlight potentially meaningful effects, we inspected and reported heatmaps only when at least one correlation exceeded an absolute threshold of |r| > 0.5.

We further examined the structure of relationships between extracted features and emotional states using principal component analysis (PCA) (Jollife and Cadima, 2016). We applied PCA to the feature-emotion correlation matrix to identify dominant patterns of association between physiological features and emotional dimensions. This approach enables dimensionality reduction at the level of interaction-driven relationships, rather than raw signal variability. Feature scores and emotion loadings were projected onto the first two principal components, which together capture the dominant patterns of variance in the correlation structure. Biplots were used to visualize clustering among features with similar emotion-related profiles, highlighting the relative contributions of the arousal and valence dimensions across perspectives.

### Statistical analysis

We performed statistical analysis to evaluate group differences and relationships between features from multiple perspectives. In this study, groups were considered to be regions of positive and negative coupling, with their corresponding feature values and emotional states. A Kruskal-Wallis test was applied to assess whether there were significant differences between groups without assuming normality, making it suitable for non-parametric data (McKight and Najab, 2010). The significance of the Pearson correlation was assessed using a *t*-test on the correlation coefficient, with the resulting *p*-value indicating whether the observed association was statistically meaningful (Komaroff, 2020). Significance was defined as a *p*-value below 0.05; however, we also allowed the threshold to drop to 0.1 for further interpretation. Statistical analyses were performed on aggregated data across segments to emphasize overall patterns rather than individual time-point inference.

## Results

### Analysis of bidirectional coupling

**FIGURE 3:**
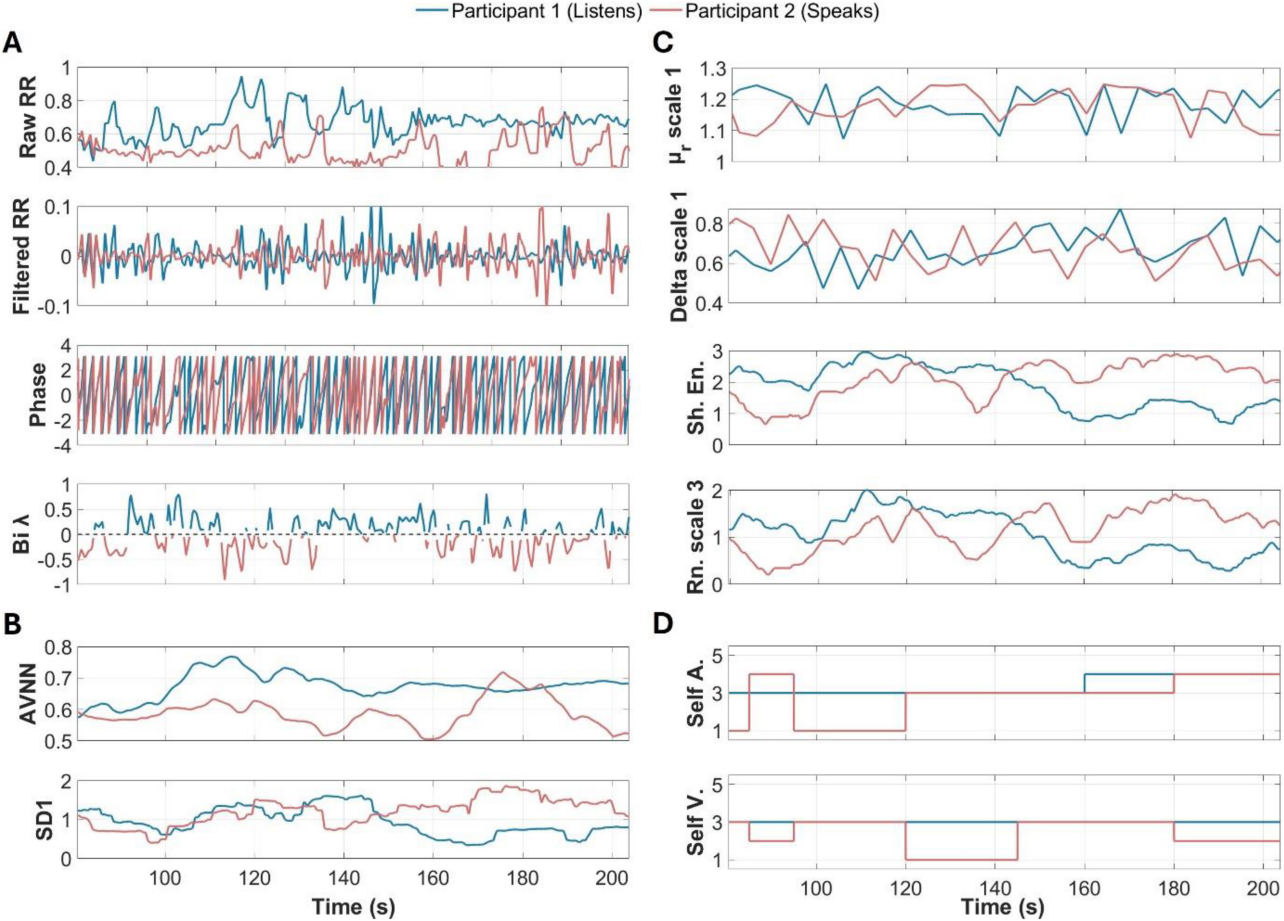
Example of the bidirectional coupling analysis at the simultaneous phase for one segment between peak-to-peak (RR) intervals. The plots show variation in phase and bidirectionality in coupling (λ) and their corresponding variation in extracted features and emotion states for participants in simultaneous phase. Showing examples of: **(A)** raw and filtered RR intervals between two participants (listening and speaking) with phase and bidirectional coupling index, **(B)** corresponding heart rate variability (HRV) features, **(C)** entropy features, and **(D)** emotional states for self annotations.

An example of bidirectional coupling is shown in Figure 3, which illustrates a simultaneous-phase segment in which Participant 1 (blue) is listening while Participant 2 (red) is speaking. In this example, the phase angles of both participants vary over time, and the corresponding coupling strength, *i.e.*, Bi λ, fluctuates between −1 and 1. These variations enable continuous tracking of the relationship between coupling strength and features or emotion states over time, allowing comparisons based on whether the interaction exhibits positive or negative coupling. For interpretation throughout, positive Bi λ indicates that the first-indexed participant’s physiology precedes the partner’s (lead), whereas negative Bi λ indicates the converse (lag/partner lead).

### Positive coupling and correlation with emotional states

**FIGURE 4:**
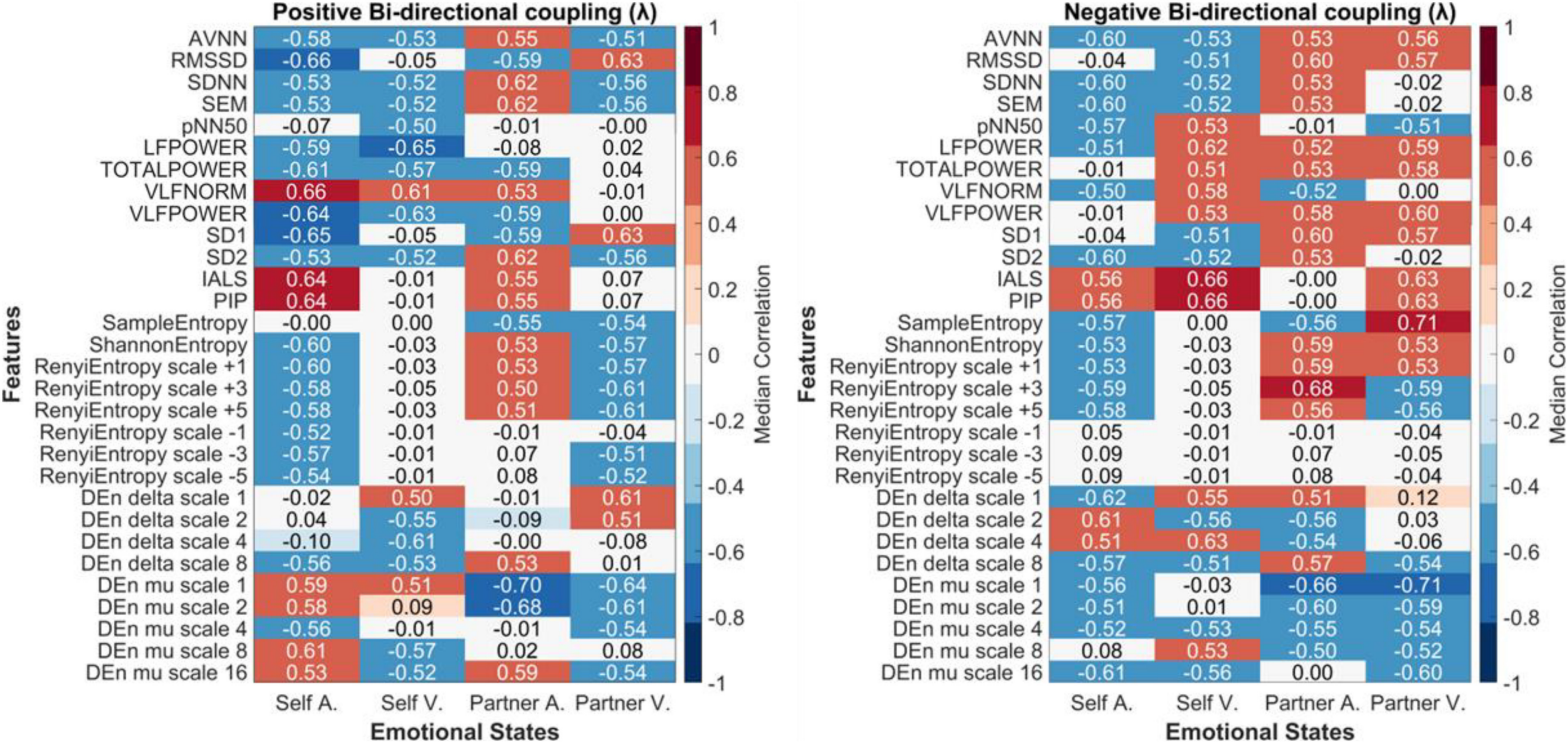
Correlation between features and emotional states at positive (left) and negative (right) bidirectional coupling (Bi λ) regions. **(Left)**: top 20 features with the highest median correlation with the four emotional states across all phases for the positive coupling region. **(Right)**: top 20 features with the highest median correlation with the four emotional states across all phases for the negative coupling region. Positive coupling indicates that the speaker is leading, while negative coupling means that the listener is leading.

The top features with the highest absolute median correlations with emotional states are shown in Figure 4. Features were aggregated across all segments within each analysis phase, along with their corresponding emotional states, based on positive and negative Bi λ regions. Among these, the majority of strong correlations in the positive coupling region (Figure 4, left side) were associated with HRV features (*n* = 13, 43.3%), with absolute values reaching nearly 0.66 (*p* < 0.001), especially for RMSSD, LF Power, and SD1. HRV features were mostly negatively correlated with self-emotions and positively correlated with partner emotions. Moreover, diffusion entropy metrics (*n* = 9, 30%), particularly μ_*r*_ and delta measures across different scales, reached absolute values of 0.70 (*p* < 0.001) for μ_*r*_ at scale 1. Here, diffusion entropy metrics were mainly positively correlated with self-emotions. The remaining 25% (*n* = 8) consisted of features such as Rényi entropy (at scales 3 and 5), Shannon entropy, and sample entropy, which had an opposite association with emotions. They were negatively correlated with self-arousal and positively correlated with partner arousal. Examples of the highest-correlating features for each emotional state are illustrated in Figure 6.

### PCA applied to positive coupling

**FIGURE 5:**
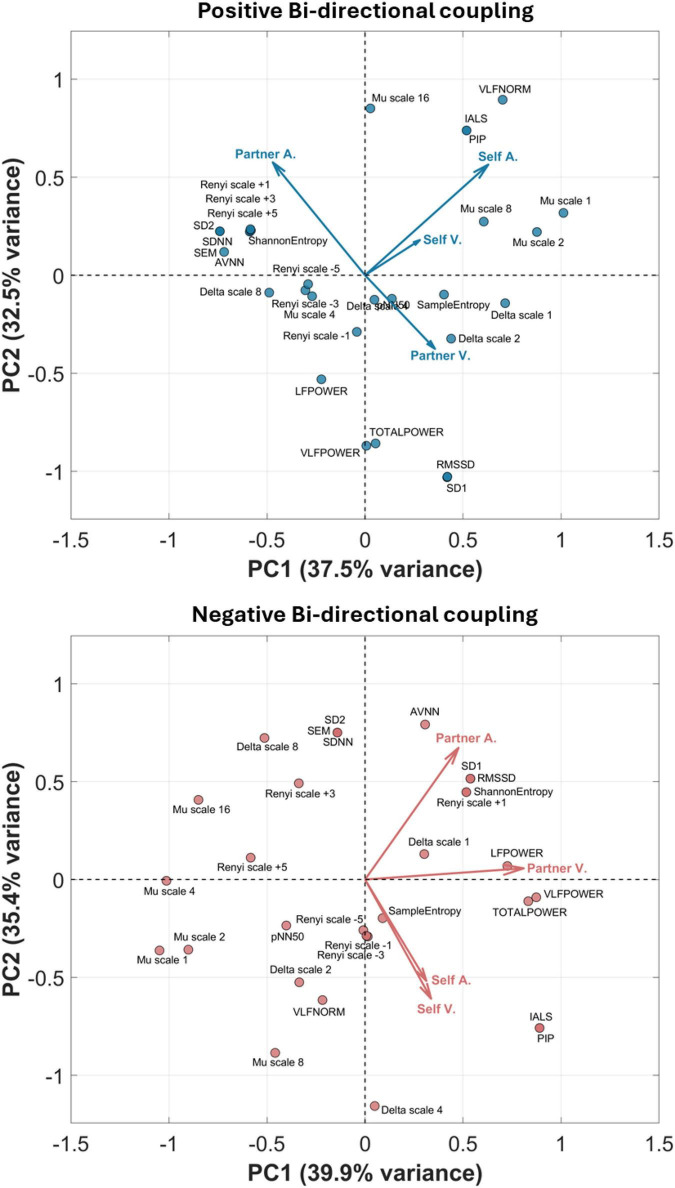
Principal component analysis (PCA) biplots of the correlations between the top 20 features and emotional states. PCA maps display how features cluster and distribute along the first two principal components, with arrows indicating emotional state vectors. Features positioned closer to the arrows reflect their correlation with emotional states. **(Top)**: results for positive bi-directional coupling, **(Bottom)**: results for negative bi-directional coupling. Positive coupling indicates that the speaker is leading, while negative coupling means that the listener is leading.

For positive coupling, the PCA biplot (Figure 5, top-side) reveals a structured separation of emotional dimensions and their associated features along the first two principal components, which together explain a substantial proportion of variance (PC1: 37.5%, PC2: 32.5%). Partner arousal loads strongly on the negative PC1 axis and clusters with time-domain HRV variability measures (AVNN, SDNN, SEM), SD2, Shannon, and Rényi entropies. In contrast, partner valence projects toward the positive PC1 and negative PC2 quadrants and aligns with power frequency measures (total power, VLF, LF) and parasympathetic indices (RMSSD, SD1). Self-arousal loads primarily on positive PC1/PC2 quadrant and is associated with μ_*r*_ features, sample entropy, and fragmentation indices (IALS, PIP). Self-valence lies closer to the origin and aligns with self arousal in characteristics.

**FIGURE 6:**
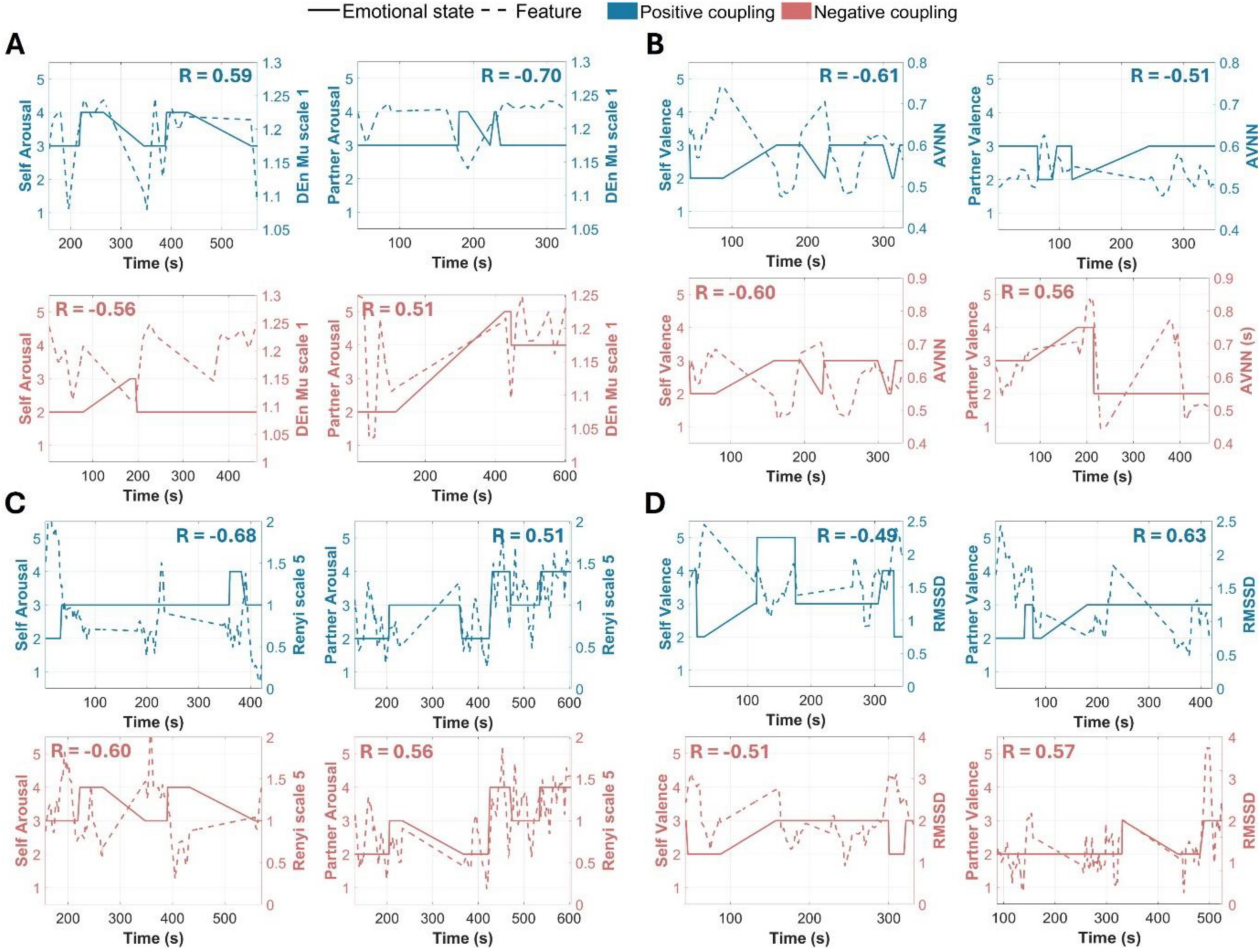
Examples of the correlation between selected features and emotional states based on positive and negative coupling. **(A)** Self and partner arousal versus diffusion entropy feature, **(B)** self and partner valence versus average normal-to-normal (AVNN) feature, **(C)** self and partner arousal versus Renyi entropy feature, and **(D)** self and partner valence versus root mean square of successive differences (RMSSD) feature. Positive coupling indicates that the speaker is leading, while negative coupling means that the listener is leading.

### Negative coupling and correlation with emotional states

On the other hand, the negative coupling regions exhibited distinct characteristics (Figure 4, right side). Here, HRV features were also prominent, with LF Power demonstrating a strong correlation with partner valence (up to 0.59, *p* < 0.001). In general, HRV features showed a pattern of correlation similar to that of positive coupling, being mostly negatively correlated with self-emotions and positively correlated with partner emotions. Notably, frequency features were positively correlated with self valence emotions. Moreover, diffusion entropy metrics showed absolute correlations of up to 0.71 (*p* < 0.001) for μ_*r*_ at scale 1, which were negatively correlated with partner emotions. Notably, Rényi entropy at scale 3 also exhibited high correlations (up to 0.68) with partner emotions, while sample entropy showed a strong positive association with partner valence, reaching 0.71 (*p* < 0.001). Examples of the highest-correlating features for each emotional state are illustrated in Figure 6.

### PCA applied to negative coupling

For negative coupling, the PCA biplot shows a distinct reorganization of emotion-feature relationships, with PC1 (39.9%) and PC2 (35.4%) jointly capturing most of the variance (Figure 5, bottom). Partner valence loads strongly on the positive PC1 axis with minimal PC2 contribution and aligns with low-frequency power. Partner arousal projects toward the positive PC1 and PC2 quadrants and clusters, as indicated by delta-time-domain HRV (SD1/SD2) and Shannon entropy. In contrast, self-related arousal and valence load toward the positive PC1 and negative PC2 quadrant, and are strongly associated with sample entropy, and negative Rényi. Notably, positive μ_*r*_ and delta concentrate in the lower PC2 region, opposing all emotional effects.

### Real and imaginary coupling associations

The comparison between the absolute (imaginary-driven) and real coupling segments reveals systematic differences in their significance in differentiating positive and negative coupling-based features (Supplementary Figure 1). Overall, the absolute coupling measure shows higher statistical significance across a broader range of features, particularly for time-domain HRV indices and power-based measures. In contrast, the real component yields fewer significant associations and lower significance levels. Moreover, AVNN and delta scale 4 were not significant at the 0.05 *p*-value threshold when using the real component, whereas pNN50, VLFNorm, PIP, IALS, and μ scale 2 showed increased statistical significance when assessed using the real component and a higher threshold at 0.001.

Moreover, PCA biplots of the correlation heatmap (Supplementary Figure 2) for the real component showed a similar, but more selective, separation of emotion-feature relationships (Supplementary Figure 3). During positive coupling, the partner’s valence loads onto positive PC2. It aligns with parasympathetic HRV indices (RMSSD, SD1) and μ_*r*_ and delta, while partner arousal associates with power-related measures (total and VLF power). Self-related arousal and valence load primarily along PC1 and align with normalized VLF and diffusion entropy features. For negative coupling, partner valence remains PC2-dominant and clusters with parasympathetic and fragmentation indices, whereas partner arousal aligns with Rényi entropy and time-domain variability. In contrast, self-arousal associates with power and negative Rényi scales, while self-valence shows weaker, intermediate associations.

### PCA applied to segments with minimal coupling

To evaluate whether the observed PCA structure reflects interaction-specific dynamics rather than intrinsic variability of physiological signals, we performed a control analysis using segments with minimal coupling (| Bi λ| < 0.1). Applying the same correlation and PCA procedures for positive and negative segments revealed that most physiological features clustered near the origin of the PCA space (Supplementary Figure 4), indicating weak and inconsistent associations with emotional states. In contrast to the structured feature-emotion organization observed during positive and negative coupling of highly correlated segments, uncoupled segments showed no clear clustering between HRV-derived features and emotional dimensions.

### Bidirectional coupling versus correlation analysis

To evaluate the proposed bidirectional coupling index relative to conventional synchrony measures, we computed time-resolved (sliding-window) Spearman correlation on the same physiological signals and segments. As shown in Supplementary Figure 5, correlation analysis revealed moderate associations between emotional states and selected features across interaction periods. However, when conditioning the analysis on coupling polarity using the bidirectional coupling index, these relationships exhibited clearer and more structured patterns.

## Discussion

This study demonstrated that bidirectional coupling of physiologically related rhythms provides a meaningful framework for characterizing interpersonal emotional dynamics during conversations. Coupling polarity in this study reflects physiological lead-lag relationships between participants’, where positive or negative coupling indicates which participant’s physiological dynamics temporally precede the other, as derived from phase relationships. While such lead-lag patterns may relate to communicative roles, e.g., positive speaker leading and negative listener leading, separating coupling into positive and negative regions showed that distinct physiological features systematically align with self- and partner-reported emotional states. The results highlight a systems neuroscience perspective in which interpersonal interaction arises from continuous brain-body coupling. The bidirectional patterns show dynamic reorganization of control, not just synchronization, and indicate active physiological synchronization involving neural, autonomic, and behavioral coordination during communication. Understanding how emotional and physiological states are continuously exchanged and regulated between individuals is crucial (Menefee et al., 2022), as these processes directly influence decision-making and conversational outcomes.

For both coupling directions, HRV emerged as a dominant correlate, particularly linking parasympathetic activity with partner-related emotions, while self-related emotions were more frequently associated with frequency power and complexity-based measures. This asymmetry supports the notion that interpersonal coupling reflects differential engagement of autonomic processes (Lettieri, 2026) depending on whether affect is internally experienced or externally perceived in the partner. It should be noted that some HRV features, particularly frequency-domain measures, are traditionally defined over longer time windows, and their interpretation in short windows within this study should be considered as relative rather than absolute. In this study, these features are used to capture short-term, time-varying fluctuations in physiological activity. As such, the resulting values reflect local spectral dynamics and should be interpreted in a comparative sense across interaction conditions. The use of shorter windows enables higher temporal resolution, allowing transient changes in autonomic activity to be aligned with conversational dynamics and interaction states.

Bidirectional coupling revealed complementary patterns that deepen understanding of the physiological response during conversations. Positive Bi λ coupling, reflecting instances when the first participant leads, was characterized by stronger associations with diffusion entropy metrics and HRV features such as RMSSD and SD1. This indicates that exerting influence over the interaction is supported by enhanced autonomic regulation and increased multiscale complexity, pointing to the physiological effort required to maintain control (Li et al., 2025). In contrast, periods of negative Bi λ coupling, indicating that the partner’s physiological dynamics lead those of the first participant, were associated with distinctive physiological signatures, including stronger correlations with Rényi entropy and higher sample entropy. These patterns suggest that when the partner drives the interaction, the first participant’s physiological system becomes more variable and complex, reflecting adaptive adjustments to externally driven dynamics (Tenev et al., 2025). In contrast, the PCA results further illustrated that partner arousal and valence consistently occupied distinct regions of the feature space compared with self-emotions, indicating that coupling polarity modulates how physiological variability, power, and complexity encode interpersonal affective exchange (Timmons et al., 2015).

It is important to note that the bidirectional coupling index contains two complementary components: the magnitude, which reflects the strength of phase synchronization between participants, and the sign (polarity), which indicates the direction of the lead-lag relationship (Allefeld and Haynes, 2022). In the present study, we focused primarily on the polarity of the coupling to distinguish speaker-leading and listener-leading interaction regimes and examine how these directional dynamics relate to emotional and physiological features during conversation. The magnitude component therefore could provide additional contextual information about synchronization strength but was not the primary focus of the present analysis. Our results show that coupling direction matters, as coupling polarity segregates autonomic regulation (vagal HRV) from complexity-based adaptation and differentiates self- versus partner-perceived effect (Gordon, 2025; Konrad et al., 2024). This strengthens the system-level account of conversation as a dynamic control process in which dyads continuously renegotiate leadership and responsiveness rather than maintaining a single stable coordination mode (Boukarras et al., 2025; Fu et al., 2021).

Our decomposition-based approach is consistent with a broader principle in physiological time-series science: classification and interpretability improve when component-specific structure is preserved rather than collapsed into a single scalar summary. Cornforth et al. (2014) demonstrated that retaining distribution-sensitive information using multiscale Rényi entropy and spectrum-like representations improves discrimination of physiological states compared with conventional single-parameter metrics, particularly in autonomic neuropathy staging. Extending this logic from single-signal complexity to interpersonal interaction dynamics, our decomposition into coupling magnitude, real-part structure, and polarity/sign preserves physiologically meaningful organization, e.g., leader-follower switching and directional interaction asymmetries, that would otherwise be obscured by magnitude-only coupling.

Decomposing the coupling metric into absolute and real components clarified the relative contributions of coupling magnitude versus in-phase synchronization. While the absolute (imaginary-driven) measure was more sensitive overall and captured a wider range of significant associations, the real component highlighted a subset of features that were otherwise masked by magnitude effects. Real-part coupling emphasized physiologically meaningful alignment related to normalization, fragmentation, and scale-specific dynamics, rather than global synchrony alone (Li et al., 2023). These findings underscore the value of combining bidirectional coupling polarity with complex-valued decomposition and real part analysis to more precisely disentangle the physiological substrates of interpersonal emotional interaction.

Finally, distinguishing between temporal directionality and causal influence in the interpretation of bidirectional coupling is of a high importance. The polarity of the coupling index reflects lead-lag relationships derived from phase differences, indicating which participant’s physiological signal temporally precedes the other (Kim et al., 2013). However, this temporal precedence does not imply causality or direct influence. Establishing causal relationships would require additional modeling frameworks or experimental manipulations beyond the scope of the present study. Accordingly, terms such as “leadership” and “responsiveness” should be interpreted as descriptors of emergent temporal dynamics rather than mechanistic or intentional causation. Moreover, separating positive (speaker-leading) and negative (listener-leading) coupling segments enabled the identification of distinct emotion-feature associations that were not apparent using correlation alone. This suggests that incorporating directional information enhances the interpretability of physiological-emotional dynamics during interaction.

### Limitations

Our study has several limitations. First, although the integrated physiology-speech-emotion analysis revealed distinct interaction patterns, the sample size and experimental context were relatively limited, as interactions were drawn from a structured, debate-based dataset. As a result, this controlled setting may not fully capture the variability and complexity of naturalistic conversational exchanges. Second, although the coupling analyses identified meaningful relationships, they cannot determine causal mechanisms. Future studies incorporating experimental interventions or causal inference frameworks will be required to establish the directionality of these effects and to systematically evaluate the relative performance of different synchrony metrics across interaction contexts. Third, while the bidirectional coupling metric captures both the magnitude and polarity of phase synchronization, the present analysis focused primarily on coupling polarity. Future work could further explore how variations in coupling magnitude contribute to the strength and stability of interpersonal physiological synchronization during conversation. Fourth, although conversational interactions are shaped by multiple behavioral cues such as facial expressions, gestures, and vocal prosody, the present study focused primarily on physiological synchronization and speech timing. Future work could extend this framework by integrating multimodal behavioral descriptors to provide a more comprehensive characterization of interpersonal dynamics. Fifth, PCA on aggregated correlation matrices captures overall association patterns but may overlook moment-to-moment dynamics. Future work could incorporate time-resolved approaches to better capture fine-grained interaction dynamics. Sixth, although the present study is based on a structured debate context, the proposed bidirectional coupling framework is general and can be extended to other forms of interaction, including cooperative or spontaneous conversations. In such settings, the interpretation of coupling polarity and its relationship to emotional dynamics may differ, reflecting context-dependent interaction patterns. Future work should explore how these dynamics vary across different conversational contexts to further establish the generalizability of the framework. Seventh, the present work concentrates on dyadic interactions, which restricts the extent to which the findings can be generalized to group-based or more dynamically evolving multi-person interactions. Extending this framework to larger and more diverse datasets will be essential for advancing our understanding of physiological and emotional dynamics in complex conversational systems. Finally, because emotional annotations were sampled at 5-s intervals and temporally expanded to align with physiological signals, both modalities exhibit temporal autocorrelation. As a result, correlation values may be influenced by non-independence of adjacent samples and should be interpreted as reflecting relative patterns of association across conditions rather than independent pointwise relationships. Future work could incorporate statistical models that explicitly account for temporal dependence to further refine these analyses.

## Conclusion

In conclusion, this study demonstrates that bidirectional phase coupling provides a powerful framework for characterizing interpersonal emotional and physiological dynamics during conversation. By integrating coupling polarity, correlation analysis, and PCA, we reveal distinct self- and partner-related affective signatures and highlight complementary roles of coupling magnitude and in-phase synchronization. Together, these findings advance understanding of how physiological synchronization supports emotional exchange in social interaction, promoting continuous conversational assessment of physiology, emotions, and speech.

## Data Availability

Publicly available datasets were analyzed in this study. This data can be found here: the K-EmoCon dataset can be accessed upon approval on https://zenodo.org/records/3762962. Access is subject to respective terms and conditions for usage.

## References

[B1] Alhussein G. Alkhodari M. Hadjileontiadis L. J. (2025). Machine learning identifies the emotion climate during naturalistic conversations using speech features and affect dynamics. *Hum. Behav. Emerg. Technol.* 2025:1915978. 10.1155/hbe2/1915978

[B2] Alhussein G. Alkhodari M. Khandoker A. H. Hadjileontiadis L. J. (2023). “Deep bispectral analysis of conversational speech towards emotional climate recognition,” in *Proceedings of the 5th IEEE International Conference on Artificial Intelligence in Engineering and Technology, IICAIET*, Kota Kinabalu, 170–175. 10.1109/IICAIET59451.2023.10291940

[B3] Alhussein G. Alkhodari M. Khandoker A. H. Hadjileontiadis L. J. (2025). Novel speech-based emotion climate recognition in peers’ conversations incorporating affect dynamics and temporal convolutional neural networks. *IEEE Access* 13 16752–16769. 10.1109/ACCESS.2025.3529125

[B4] Alkhodari M. Apostolidis G. Zisou C. Hadjileontiadis L. J. Khandoker A. H. (2021a). “Swarm decomposition enhances the discrimination of cardiac arrhythmias in varied-lead ECG using ResNet-BiLSTM network activations,” in *Proceedings of the 2021 Computing in Cardiology (CinC)*, (Brno: IEEE).

[B5] Alkhodari M. Jelinek H. F. Saleem S. Hadjileontiadis L. J. Khandoker A. H. (2021b). Revisiting left ventricular ejection fraction levels: A circadian heart rate variability-based approach. *IEEE Access* 9 130111–130126. 10.21203/rs.3.rs-367988/v1 36284789

[B6] Alkhodari M. Khandoker A. H. Jelinek H. F. Karlas A. Soulaidopoulos S. Arsenos P. et al. (2024). Circadian assessment of heart failure using explainable deep learning and novel multi-parameter polar images. *Comput. Methods Programs Biomed.* 248:108107. 10.1016/j.cmpb.2024.108107 38484409

[B7] Allefeld C. Haynes J. D. (2022). Multistage classification identifies altered cortical phase- and amplitude-coupling in multiple sclerosis. *Neuroimage* 264:119752. 10.1016/j.neuroimage.2022.119752 36400377 PMC9771829

[B8] Antonelli F. Bernardi F. Koul A. Novembre G. Papaleo F. (2025). Emotions in multi-brain dynamics: A promising research frontier. *Neurosci. Biobehav. Rev.* 168:105965. 10.1016/j.neubiorev.2024.105965 39617219

[B9] Armañac-Julián P. Kontaxis S. Lázaro J. Rapalis A. Brazaitis M. Marozas V. et al. (2025). Vascular reactivity characterized by PPG-derived pulse wave velocity. *Biomed. Signal Process. Control* 105:107641. 10.1016/j.bspc.2025.107641

[B10] Benfatto M. Pace E. Curceanu C. Scordo A. Clozza A. Davoli I. et al. (2021). Biophotons and emergence of quantum coherence-a diffusion entropy analysis. *Entropy* 23:554. 10.3390/e23050554 33947077 PMC8146849

[B11] Birchler G. R. Weiss R. L. Vincent J. P. (2024). Two’s company: Biobehavioral research with dyads. *Biol. Psychol.* 185:108719. 10.1016/j.biopsycho.2023.108719 37939868

[B12] Boukarras S. Placidi V. Rossano F. Era V. Aglioti S. M. Candidi M. et al. (2025). Interpersonal physiological synchrony during dyadic joint action is increased by task novelty and reduced by social anxiety. *Psychophysiology* 62:e70031. 10.1111/psyp.70031 40097345 PMC11913774

[B13] Charlton P. H. Bonnici T. Tarassenko L. Alastruey J. Clifton D. A. Beale R. et al. (2017). Extraction of respiratory signals from the electrocardiogram and photoplethysmogram: Technical and physiological determinants. *Physiol. Meas.* 38 669–690. 10.1088/1361-6579/aa670e 28296645

[B14] Chatterjee I. Goršiè M. Clapp J. D. Novak D. (2021). Automatic estimation of interpersonal engagement during naturalistic conversation using dyadic physiological measurements. *Front. Neurosci.* 15:757381. 10.3389/fnins.2021.757381 34764854 PMC8576061

[B15] Chatterjee I. Goršiè M. Kaya R. A. Clapp J. D. Novak V. D. (2025). Estimating the valence and arousal of dyadic conversations using autonomic nervous system responses and regression algorithms. *Front. Neuroergon.* 6:1671311. 10.3389/fnrgo.2025.1671311 41416252 PMC12708908

[B16] Cornforth D. J. Tarvainen M. P. Jelinek H. F. (2013). Using renyi entropy to detect early cardiac autonomic neuropathy. *Annu. Int. Conf. IEEE Eng. Med. Biol. Soc.* 2013 5562–5565. 10.1109/EMBC.2013.6610810 24110997

[B17] Cornforth D. J. Tarvainen M. P. Jelinek H. F. (2014). How to calculate Renyi entropy from heart rate variability, and why it matters for detecting cardiac autonomic neuropathy. *Front. Bioeng. Biotechnol.* 2:34. 10.3389/fbioe.2014.00034 25250311 PMC4159033

[B18] de Zambotti M. Trinder J. Silvani A. Colrain I. M. Baker F. C. (2018). Dynamic coupling between the central and autonomic nervous systems during sleep: A review. *Neurosci. Biobehav. Rev.* 90 84–103. 10.1016/j.neubiorev.2018.03.027 29608990 PMC5993613

[B19] Dikker S. Wan L. Davidesco I. Kaggen L. Oostrik M. McClintock J. et al. (2017). Brain-to-brain synchrony tracks real-world dynamic group interactions in the classroom. *Curr. Biol.* 27 1375–1380. 10.1016/j.cub.2017.04.002 28457867

[B20] Divjak D. Sun H. Milin P. (2024). Physiological responses and cognitive behaviours: Measures of heart rate variability index language knowledge. *J. Neurolinguistics* 69:101177. 10.1016/j.jneuroling.2023.101177

[B21] Feutrill A. Roughan M. (2021). A review of shannon and differential entropy rate estimation. *Entropy* 23:1046. 10.3390/e23081046 34441186 PMC8392187

[B22] Fu D. Incio-Serra N. Motta-Ochoa R. Blain-Moraes S. (2021). Interpersonal physiological synchrony for detecting moments of connection in persons with dementia: A pilot study. *Front. Psychol.* 12:749710. 10.3389/fpsyg.2021.749710 34966322 PMC8711588

[B23] Gordon I. (2025). Interpersonal synchrony research in human groups. *Soc. Personal. Psychol. Compass* 19:e70068. 10.1111/spc3.70068

[B24] Hadjileontiadis L. J. Liatsos C. N. Mavrogiannis C. C. Rokkas T. A. Panas S. M. (2000). Enhancement of bowel sounds by wavelet-based filtering. *IEEE Trans. Biomed. Eng.* 47 876–886. 10.1109/10.846681 10916258

[B25] Helm J. L. Miller J. G. Kahle S. Troxel N. R. Hastings P. D. (2018). On measuring and modeling physiological synchrony in dyads. *Multivariate Behav. Res.* 53 521–543. 10.1080/00273171.2018 29683720

[B26] Hess U. Dietrich J. Kafetsios K. Elkabetz S. Hareli S. (2020). The bidirectional influence of emotion expressions and context: Emotion expressions, situational information and real-world knowledge combine to inform observers’ judgments of both the emotion expressions and the situation. *Cogn. Emot.* 34 539–552. 10.1080/02699931.2019 31500504

[B27] Jelinek H. F. Alkhodari M. Khandoker A. H. Hadjileontiadis L. J. (2025). Oscillatory components of bidirectional cardio-respiratory coupling in depression and suicidal ideation: Insights from swarm decomposition and entropy analysis. *Front. Netw. Physiol.* 5:1620862. 10.3389/fnetp.2025.1620862 41064489 PMC12500556

[B28] Jelinek H. F. Khandoker A. H. (2020). Reducing suicidal ideation by biofeedback-guided respiration – heart rate coherence. *Digit. Psychiatry* 3 1–11. 10.1080/2575517X.2020.1732733

[B29] Jelinek H. F. Tuladhar R. Culbreth G. Bohara G. Cornforth D. West B. J. et al. (2021). Diffusion entropy vs. multiscale and rényi entropy to detect progression of autonomic neuropathy. *Front. Physiol.* 11:607324. 10.3389/fphys.2020.607324 33519512 PMC7841429

[B30] Jiang J. Chen C. Dai B. Shi G. Ding G. Liu L. et al. (2015). Leader emergence through interpersonal neural synchronization. *Proc. Natl. Acad. Sci. U. S. A.* 112 4274–4279. 10.1073/pnas.1422930112 25831535 PMC4394311

[B31] Jiang J. Zheng L. Lu C. (2021). A hierarchical model for interpersonal verbal communication. *Soc. Cogn. Affect. Neurosci.* 16 246–255. 10.1093/scan/nsaa151 33150951 PMC7812628

[B32] Jollife I. T. Cadima J. (2016). Principal component analysis: A review and recent developments. *Philos. Trans. R. Soc. A Math. Phys. Eng. Sci.* 374:20150202. 10.1098/rsta.2015.0202 26953178 PMC4792409

[B33] Kim E. Kim D. S. Ahmad F. Park H. (2013). Pattern-based granger causality mapping in fMRI. *Brain Connect.* 3 569–577. 10.1089/brain.2013.0148 24059863 PMC3868251

[B34] Komaroff E. (2020). Relationships between p-values and pearson correlation coefficients, type 1 errors and effect size errors, under a true null hypothesis. *J. Stat. Theory Pract.* 14:49. 10.1007/s42519-020-00115-6

[B35] Konrad K. Gerloff C. Kohl S. H. Mehler D. M. A. Mehlem L. Volbert E. L. et al. (2024). Interpersonal neural synchrony and mental disorders: Unlocking potential pathways for clinical interventions. *Front. Neurosci.* 18:1286130. 10.3389/fnins.2024.1286130 38529267 PMC10962391

[B36] Konvalinka I. Bauer M. Stahlhut C. Hansen L. K. Roepstorff A. Frith C. D. et al. (2014). Frontal alpha oscillations distinguish leaders from followers: Multivariate decoding of mutually interacting brains. *Neuroimage* 94 79–88. 10.1016/j.neuroimage.2014.03.003 24631790

[B37] Lapitan D. G. Rogatkin D. A. Molchanova E. A. Tarasov A. P. (2024). Estimation of phase distortions of the photoplethysmographic signal in digital IIR filtering. *Sci. Rep.* 14:6546. 10.1038/s41598-024-57297-3 38503856 PMC10951216

[B38] Leiva K. M. R. Vondal M. Yang M. Cerny M. Wang Y. (2025). Estimating respiratory rate in freely moving users using independent component and multi-resolution analysis. *Comput. Biol. Med.* 197:110957. 10.1016/j.compbiomed.2025.110957 40865266

[B39] Lettieri G. (2026). Integrating neural, physiological, and interoceptive measures in social interaction. *Front. Neurosci.* 20:1771470. 10.3389/fnins.2026.1771470 41808897 PMC12968177

[B40] Li C. Liu S. Wang Z. Yuan G. (2023). Classifying epileptic phase-amplitude coupling in SEEG using complex-valued convolutional neural network. *Front. Physiol.* 13:1085530. 10.3389/fphys.2022.1085530 36685186 PMC9849379

[B41] Li L. Liang S. Bai J. Zeng Y. Zhang M. Li Z. et al. (2025). Regulation of autonomic nervous system by acupuncture: A heart rate variability study on physical stress. *Front. Hum. Neurosci.* 19:1676863. 10.3389/fnhum.2025.1676863 41307072 PMC12644030

[B42] Lin D. Zhu T. Wang Y. (2024). Emotion contagion and physiological synchrony: The more intimate relationships, the more contagion of positive emotions. *Physiol. Behav.* 275:114434. 10.1016/j.physbeh.2023.114434 38092069

[B43] McKight P. E. Najab J. (2010). Kruskal-Wallis test. *Corsini Encycl. Psychol.* 1 1–10. 10.1002/9780470479216.CORPSY0491

[B44] Menefee D. S. Ledoux T. Johnston C. A. (2022). The importance of emotional regulation in mental health. *Am. J. Lifestyle Med.* 16 28–31. 10.1177/15598276211049771 35185423 PMC8848120

[B45] Nasrat S. A. Mahmoodi K. Khandoker A. H. Grigolini P. Jelinek H. F. (2023). Multiscale diffusion entropy analysis for the detection of crucial events in cardiac pathology. *Annu. Int. Conf. IEEE Eng. Med. Biol. Soc.* 2023:10340403. 10.1109/EMBC40787.2023.10340403 38083786

[B46] Park C. Y. Cha N. Kang S. Kim A. Khandoker A. H. Hadjileontiadis L. et al. (2020). K-EmoCon, a multimodal sensor dataset for continuous emotion recognition in naturalistic conversations. *Sci. Data* 7:293. 10.1038/s41597-020-00630-y 32901038 PMC7479607

[B47] Patel P. Raghunandan R. Annavarapu R. N. (2021). EEG-based human emotion recognition using entropy as a feature extraction measure. *Brain Inform.* 8:20. 10.1186/s40708-021-00141-5 34609639 PMC8492873

[B48] Saleem S. Khandoker A. H. Alkhodari M. Hadjileontiadis L. J. Jelinek H. F. (2022). A two-step pre-processing tool to remove Gaussian and ectopic noise for heart rate variability analysis. *Sci. Rep.* 12:18396. 10.1038/s41598-022-21776-2 36319659 PMC9626590

[B49] Schoot L. Hagoort P. Segaert K. (2016). What can we learn from a two-brain approach to verbal interaction? *Neurosci. Biobehav. Rev.* 68 454–459. 10.1016/j.neubiorev.2016.06.009 27311632

[B50] Stam C. J. Nolte G. Daffertshofer A. (2007). Phase lag index: Assessment of functional connectivity from multi channel EEG and MEG with diminished bias from common sources. *Hum. Brain Mapp.* 28 1178–1193. 10.1002/hbm.20346 17266107 PMC6871367

[B51] Subakan C. Ravanelli M. Cornell S. Grondin F. Bronzi M. (2023). Exploring self-attention mechanisms for speech separation. *IEEE/ACM Trans. Audio Speech Lang. Process.* 31 2169–2180. 10.1109/TASLP.2023.3282097

[B52] Tenev A. Markovska-Simoska S. Müller A. Mishkovski I. (2025). Entropy, complexity, and spectral features of EEG signals in autism and typical development: A quantitative approach. *Front. Psychiatry* 16:1505297. 10.3389/fpsyt.2025.1505297 39967584 PMC11832502

[B53] Timmons A. C. Margolin G. Saxbe D. E. (2015). Physiological linkage in couples and its implications for individual and interpersonal functioning: A literature review. *J. Fam. Psychol.* 29 720–731. 10.1037/fam0000115 26147932 PMC4593729

[B54] Tschacher W. Meier D. (2020). Physiological synchrony in psychotherapy sessions. *Psychother. Res.* 30 558–573. 10.1080/10503307.2019 31060474

[B55] Vinck M. Oostenveld R. Van Wingerden M. Battaglia F. Pennartz C. M. A. (2011). An improved index of phase-synchronization for electrophysiological data in the presence of volume-conduction, noise and sample-size bias. *Neuroimage* 55 1548–1565. 10.1016/j.neuroimage.2011.01.055 21276857

[B56] Wang L. Hao J. Zhou T. H. (2023). ECG multi-emotion recognition based on heart rate variability signal features mining. *Sensors* 23:8636. 10.3390/s23208636 37896729 PMC10610830

[B57] Ziogas I. Alfalahi H. Khandoker A. H. Hadjileontiadis L. J. (2024). “Cochceps-augment: A novel self-supervised contrastive learning using cochlear cepstrum-based masking for speech emotion recognition,” in *Proceedings of the 2024 IEEE International Conference on Acoustics, Speech, and Signal Processing Workshops (ICASSPW)*, (Seoul: IEEE), 700–704. 10.1109/ICASSPW62465.2024.10626164

