# Supplementary material for: Continuous bidirectional coupling of heart rate variability and emotions in dyad speaker-listener dynamics reveals unique interpersonal synchronization
Source: Front Syst Neurosci. 2026 May 29;20:1802891. doi: 10.3389/fnsys.2026.1802891 (PMC13261717; doi:10.3389/fnsys.2026.1802891)
Supplement: Supplementary file 1 [file Data_Sheet_1.docx]

**
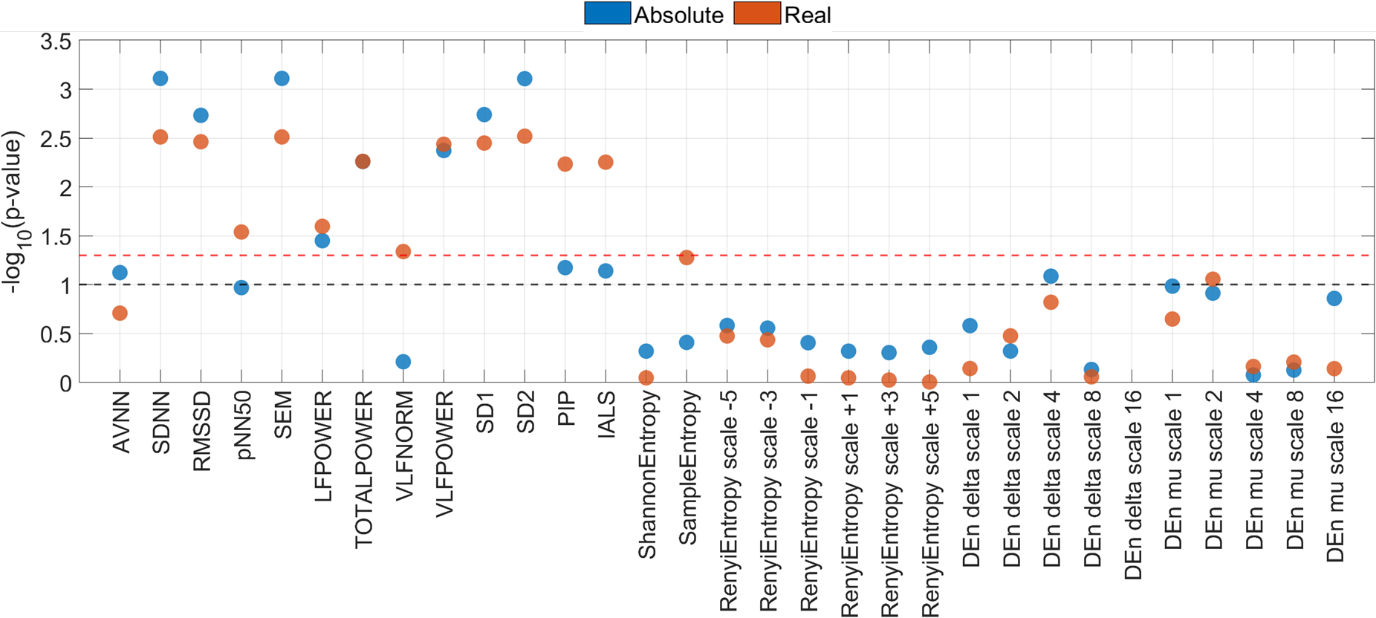
Supplementary Figure 1. Statistical analysis of differences between positive and negative coupling feature segments for the original (absolute) and real-part bidirectional coupling.**


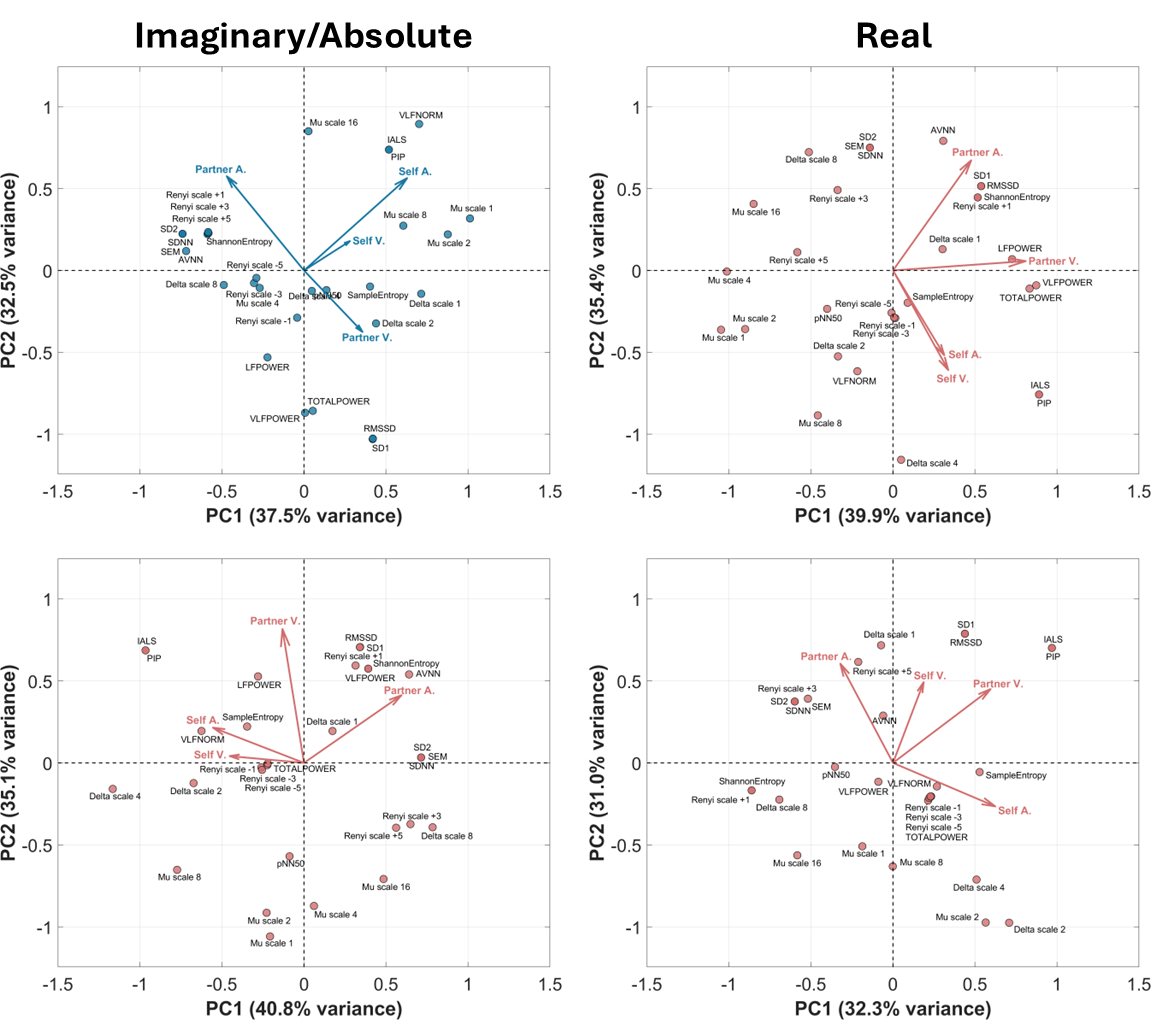
**Supplementary Figure 2. Principal component analysis (PCA) biplots of the correlations between top features and emotional states for positive (top) and negative (bottom) coupling in comparison with the real part coupling.** Positive coupling indicates that the speaker is leading, while negative coupling means that the listener is leading.


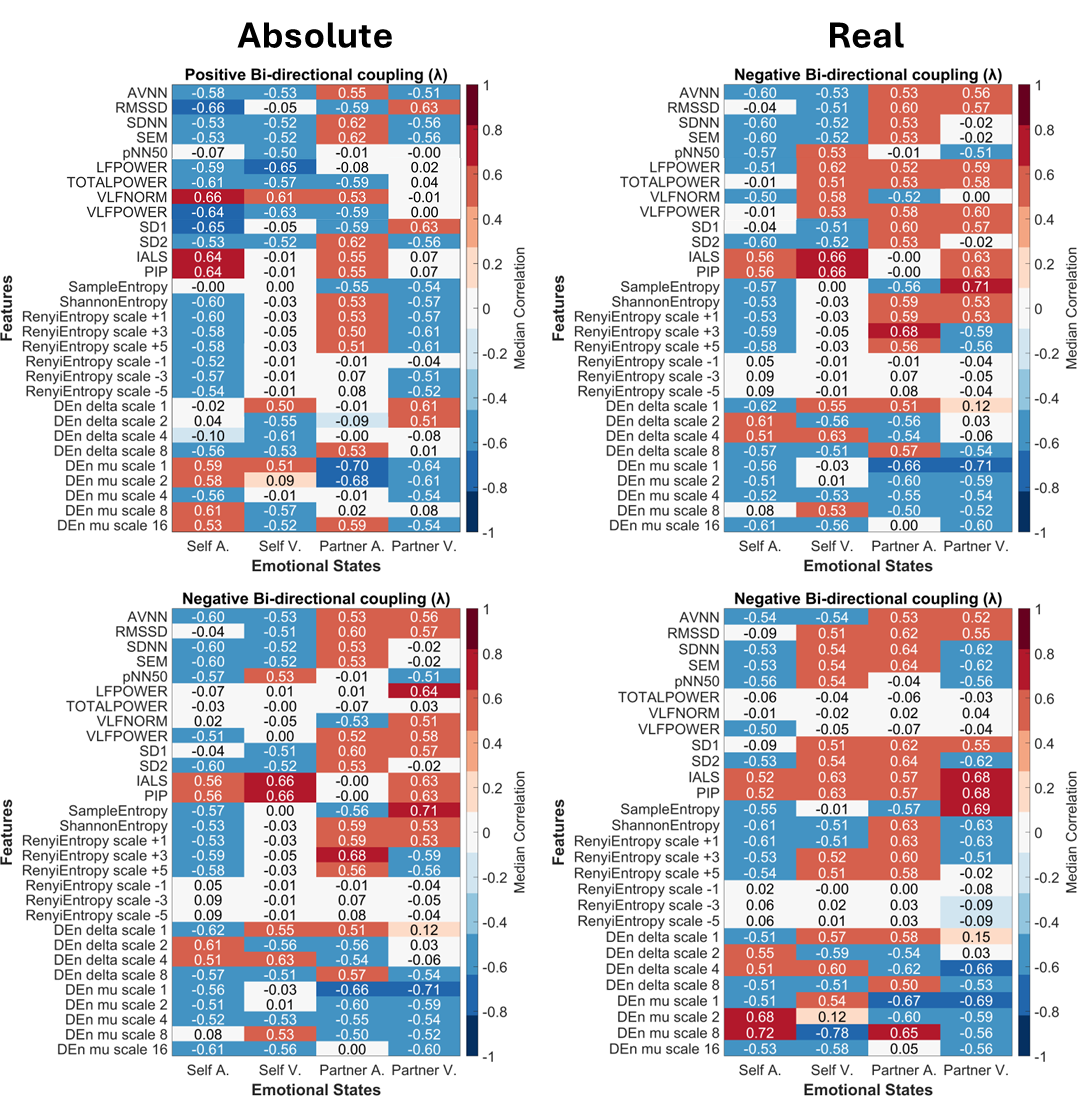
**Supplementary Figure 3. Correlation between features and emotional states at positive (top) and negative (bottom) bidirectional coupling (Bi λ) regions in comparison with the real part coupling.** Positive coupling indicates that the speaker is leading, while negative coupling means that the listener is leading.

**
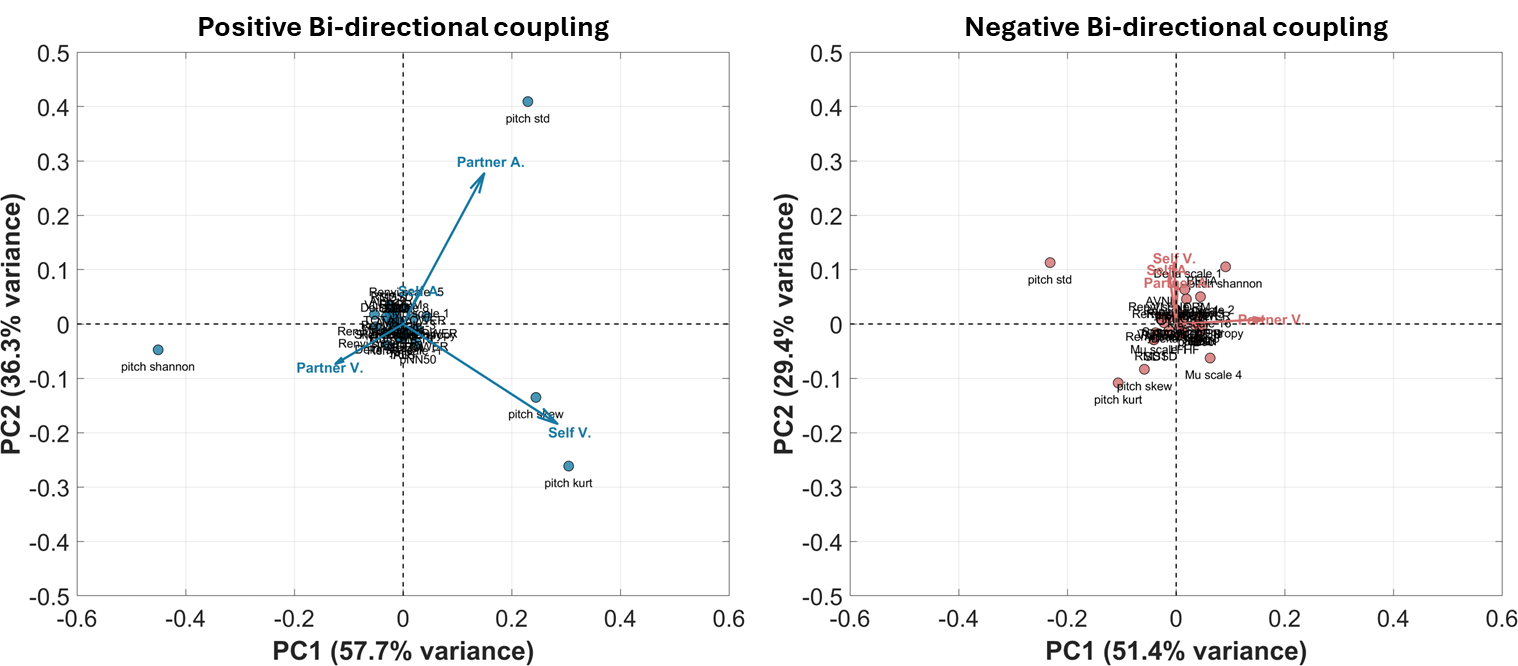
Supplementary Figure 4. Principal component analysis (PCA) biplots of the nearly no correlations (below 0.1) of the top 20 features and emotional states.** Positive coupling indicates that the speaker is leading, while negative coupling means that the listener is leading.


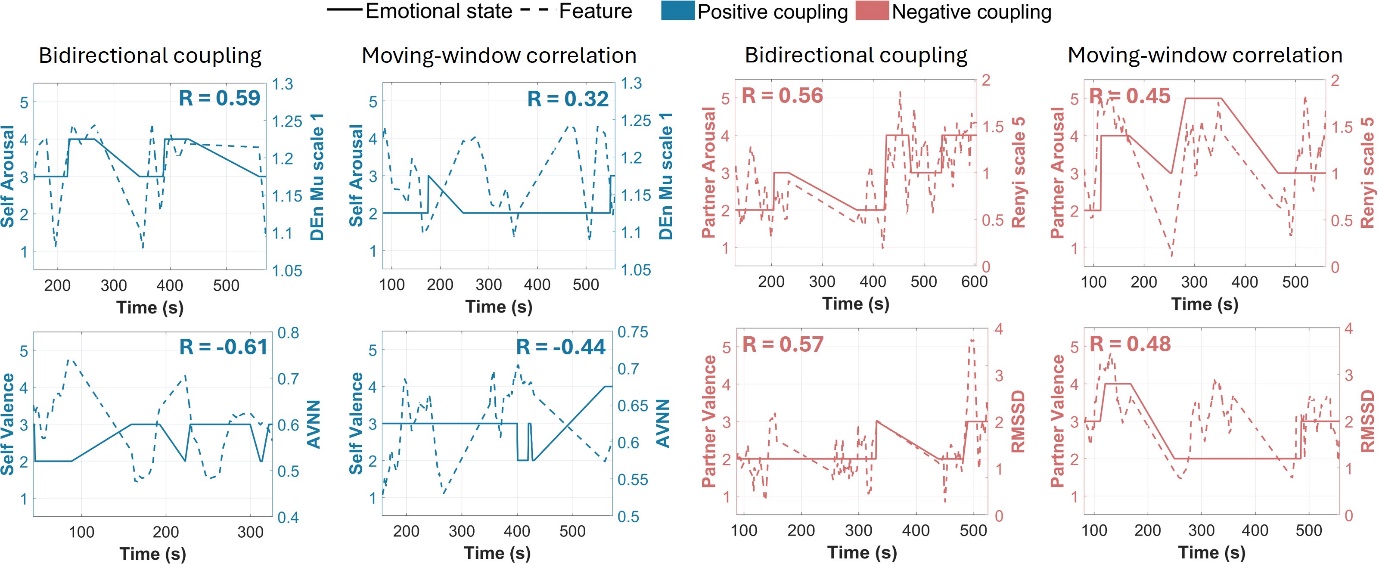
**Supplementary Figure 5. Comparison between the bidirectional coupling index and moving-window Pearson correlation.** Positive coupling indicates that the speaker is leading, while negative coupling means that the listener is leading.
